# Characterization of developmental defects in the forebrain resulting from hyperactivated mTOR signaling by integrative analysis of transcriptomic and proteomic data

**DOI:** 10.1038/s41598-017-02842-6

**Published:** 2017-06-06

**Authors:** Jiheon Shin, Minhyung Kim, Hee-Jung Jung, Hye Lim Cha, Haeyoung Suh-Kim, Sanghyun Ahn, Jaehoon Jung, YounAh Kim, Yukyung Jun, Sanghyuk Lee, Daehee Hwang, Jaesang Kim

**Affiliations:** 1 Department of Life Science, Ewha Womans University, Seoul, 03760 Republic of Korea; 2 Ewha Research Center for Systems Biology, Ewha Womans University, Seoul, 03760 Republic of Korea; 3 School of Interdisciplinary Bioscience and Bioengineering, Pohang University of Science and Technology, Pohang, 37666 Republic of Korea; 4 Center for Plant Aging Research, Institute for Basic Science, Daegu Gyeongbuk Institute of Science and Technology (DGIST), Daegu, 42988 Republic of Korea; 5 Department of Anatomy, Ajou University School of Medicine, Suwon, 16499 Republic of Korea; 6 Department of Biomedical Sciences, The Graduate School, Ajou University School of Medicine, Suwon, 16499 Republic of Korea; 7 Department of New Biology, DGIST, Daegu, 42988 Republic of Korea; 8 Ewha-JAX Research Center for Cancer Immunotherapy, Ewha Womans University, Seoul, 03760 Korea

## Abstract

Hyperactivated mTOR signaling in the developing brain has been implicated in multiple forms of pathology including tuberous sclerosis complex (TSC). To date, various phenotypic defects such as cortical lamination irregularity, subependymal nodule formation, dysmorphic astrocyte differentiation and dendritic malformation have been described for patients and animal models. However, downstream networks affected in the developing brain by hyperactivated mTOR signaling have yet to be characterized. Here, we present an integrated analysis of transcriptomes and proteomes generated from wild-type and Tsc1/Emx1-Cre forebrains. This led to comprehensive lists of genes and proteins whose expression levels were altered by hyperactivated mTOR signaling. Further incorporation of TSC patient data followed by functional enrichment and network analyses pointed to changes in molecular components and cellular processes associated with neuronal differentiation and morphogenesis as the key downstream events underlying developmental and morphological defects in TSC. Our results provide novel and fundamental molecular bases for understanding hyperactivated mTOR signaling-induced brain defects which can in turn facilitate identification of potential diagnostic markers and therapeutic targets for mTOR signaling-related neurological disorders.

## Introduction

Mechanistic target of rapamycin (mTOR) signaling is involved in a broad spectrum of brain development processes including proliferation of neural stem cells, differentiation of neurons, and assembly and maintenance of neuronal circuits^1^. mTOR signaling also functions in regulation of essential neurophysiological behaviors, such as sleep, feeding, and circadian rhythm^1^. Not surprisingly, dysregulation of mTOR signaling is implicated in diverse neurological disorders encompassing developmental, degenerative and psychiatric types. Most notable among them is tuberous sclerosis complex (TSC) which is an autosomal dominant disease caused by mutations of TSC1 and TSC2 genes respectively encoding hamartin and tuberin proteins^2, 3^. Loss-of-function of these genes, which act as negative regulators of mTOR signaling, leads to hyperactivation of the signaling pathway. TSC leads to formation of benign tumors in multiple organs including brain, kidney, lung, and skin^2, 3^. Abnormal brain development featuring such benign tumors is often observed in TSC patients, resulting in intellectual disability, epileptic seizures, and autism^2, 3^.

A murine TSC model with brain-specific hyperactivation of mTOR signaling pathway can be obtained via the use of Emx1-Cre mouse line in combination with loxP-TSC1 line as previously described^4, 5^. Magri *et al*.^4^ reported that this model partially recapitulates TSC with lesions including cortical lamination defects and subependymal nodule formation. Carson *et al*.^5^ also reported that this model shows the cortical lamination defect as a prominent phenotype as well as enlarged dysmorphic astrocytes and decreased myelination. Notably, prenatal treatments with rapamycin, an mTOR inhibitor, rescued these mice from brain malformation and early death, indicating that hyperactivated mTOR signaling is indeed responsible for the aberrant phenotypes. Moreover, hyperactivation of mTOR signaling has also been examined for its effects on neuronal development *in vitro* where neuronal morphology and property could be examined in detail^6, 7^. Consistent with the observation *in vivo*, loss of TSC1 or TSC2 triggered enlargement of neuronal somas. Further, dendritic morphology showed clear alterations and decreased density along the dendrites. In addition, Choi *et al*.^7^ reported that depletion of TSC1 or TSC2 induced formation of extra axons. Together, these data indicate that mTOR signaling is critically involved in neuronal maturation process including cell body growth, axonogenesis, and synaptogenesis.

There have been only limited efforts to characterize genes and/or proteins underlying such phenotypic consequences of the hyperactivated mTOR signaling despite the clear effects observed both *in vivo* and *in vitro*. Nie *et al*.^8^ performed gene expression profiling of hippocampal neurons after knockdown of TSC2 and found that expression of Atf3, a transcription factor (TF), and of Gelsolin (GSN), one of its target genes with a role in regulating actin cytoskeleton, is up-regulated. They also showed that shRNA-mediated down-regulation of Atf3 not only reduces Gelsolin expression but also rescues dendritic spine defects, thereby providing a set of downstream effectors of mTOR signaling in developing neurons. Clearly, this represents only a minor fraction of the molecular network downstream to mTOR signaling, especially given that characterization of developing brain has yet to be carried out.

Here, we describe an integrated analysis of transcriptomes and proteomes generated from telencephalon tissues of the forebrain in Tsc1/Emx1-Cre mice. Comparative analyses of data from wild-type (WT) and Tsc1/Emx1-Cre telencephalons revealed sets of genes and proteins altered by TSC1 knockout. Moreover, functional enrichment analyses of these genes and proteins together with gene expression data from TSC patients further prioritized important downstream components and their associated pathways, providing a core molecular network affected by TSC1-dependent hyperactivation of mTOR signaling. In sum, this study establishes fundamental molecular bases for phenotypic aberrations caused by hyperactivated mTOR signaling in the brain, which can be useful for isolation of biomarkers and/or therapeutic targets of mTOR signaling-related neurological disorders.

## Results

### Transcriptomic and proteomic analysis of forebrain tissues with hyperactivated mTOR signaling

We first performed mRNA-sequencing in order to obtain gene expression profiles of telencephalon tissues in the forebrain. Three independent WT (n = 3) and Tsc1/Emx1-Cre (TSC1) mice (n = 3) at postnatal day 0 were used for the sequencing (Materials and Methods). From the mRNA-sequencing, 125.3 million reads on average were obtained from individual samples, and aligning them to the mouse genome resulted in 10.1 Giga bps of mapped sequences corresponding to 120.2-fold coverage of the annotated mouse transcriptome (Materials and Methods). In the six samples, 14,396 genes on average were found to be expressed (Materials and Methods).

**Figure 1:**
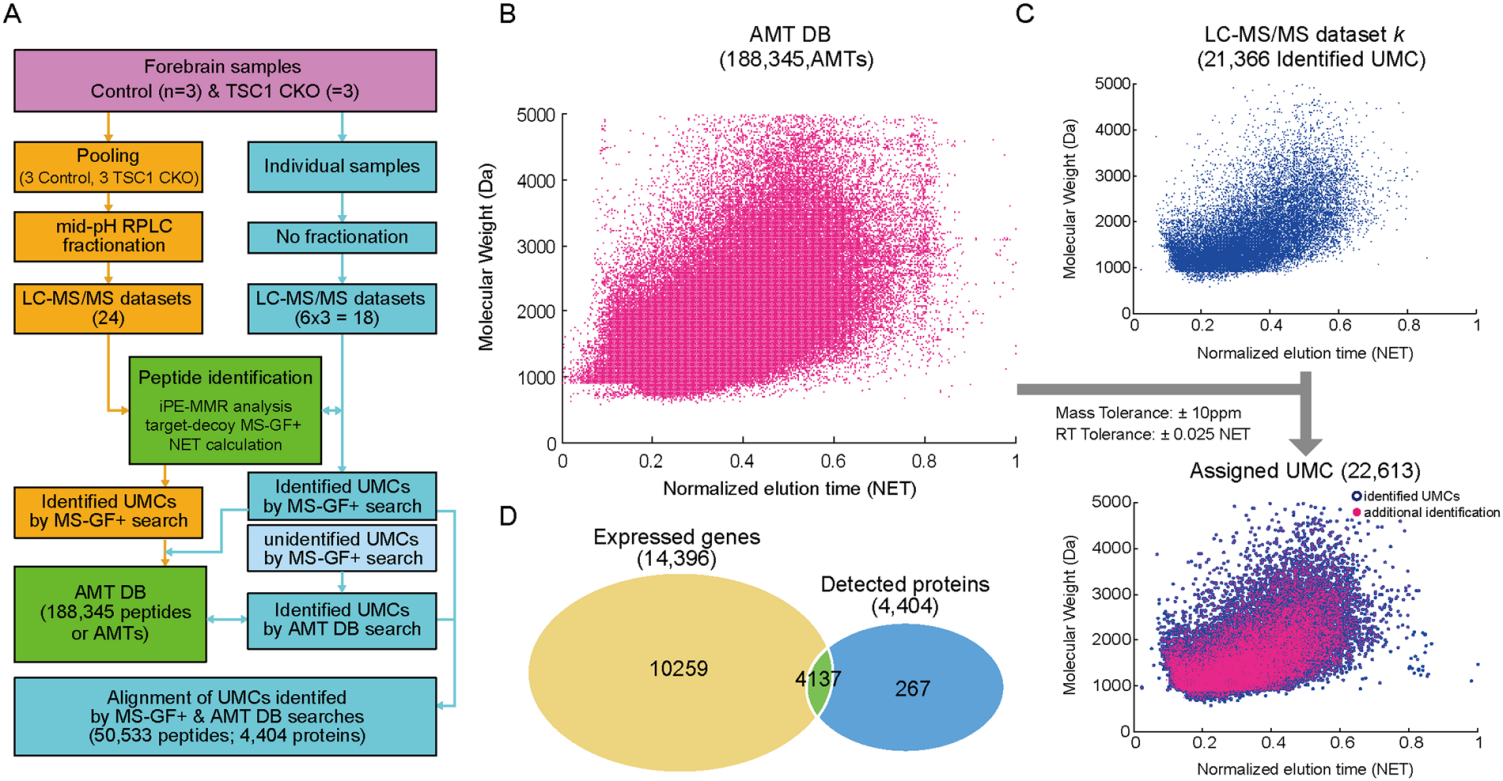
The master AMT DB. (**A**) The overall scheme of AMT DB construction. A total of 42 datasets (24 + 18 from left and right branches, respectively) were generated from LC-MS/MS analysis. For individual datasets, UMCs assigned with protein IDs (identified UMCs) were identified using *i*PE-MMR analysis and target-decoy MS-GF+ search and then used to construct the AMT DB. (**B**,**C**) Utilization of AMT DB to assign protein IDs to unidentified UMCs. The 188,345 AMTs (magenta dots) in the AMT DB are visualized in a 2D (NET and molecular weight) scatter plot (**B**). For a LC-MS/MS dataset (dataset *k*), the identified UMCs (blue dots) are shown in the upper scatter plot. By matching unidentified UMCs in this dataset with AMTs using the indicated mass and NET tolerances, a subset of unidentified UMCs (magenta dots) were assigned with protein IDs. These matched UMCs are shown in the bottom scatter plot (**C**). (**D**) Relationships of expressed genes identified from mRNA-sequencing data with detected proteins from LC-MS/MS datasets. Numbers in parentheses denote the numbers of expressed genes and detected proteins.

Next, for a comprehensive proteome profiling of forebrains with hyperactivated mTOR signaling, we generated a master accurate mass and time tag (AMT) database (DB) as previously described^9^. To this end, we first generated peptides using the filter-aided sample preparation (FASP) method for protein samples independently obtained from three WT (n = 3) and three TSC1 (n = 3) mice. After pooling peptides from individual samples, we performed mid-pH reverse phase LC (RPLC)-based fractionation of the pooled peptide sample into 24 fractions (Fig. 1A, left branch; Materials and Methods). The 24 fractions were then subjected to liquid chromatography-tandem mass spectrometry (LC-MS/MS) analysis, resulting in 24 LC-MS/MS datasets (Materials and Methods). In addition, we performed triplicate experiments for the peptide samples from each of the three WT and three TSC1 mice, resulting in 18 (3 × 6) LC-MS/MS datasets (Fig. 1A, right branch).

We then generated unique mass classes (UMCs) of peptide MS features from the 42 (24 + 18) LC-MS/MS datasets using iPE-MMR analysis^10^, assigned peptide IDs and normalized elution times (NETs) to UMCs (identified UMCs) after target-decoy MS-GF+ search (false discovery rate <0.01) and NET calculation. All identified UMCs were compiled into the AMT DB that comprised 188,345 peptides (Fig. 1A, AMT DB; Fig. 1B; Materials and Methods). For each dataset, we assigned peptide IDs to unidentified UMCs using the AMT DB by matching them with AMTs within the mass and NET tolerances of 10 ppm and 0.025 NET, respectively (magenta dots for additionally identified UMCs by AMT DB in Fig. 1C, bottom). By combining the MS-GF+ and AMT DB search results, 50,533 peptides were identified from 18 LC-MS/MS datasets, corresponding to 4,404 proteins. Of the 4,404 detected proteins, 4,137 overlapped with the 14,396 expressed genes identified from the mRNA-sequencing (Fig. 1D).

### Genes and proteins affected by hyperactivated mTOR signaling

**Figure 2:**
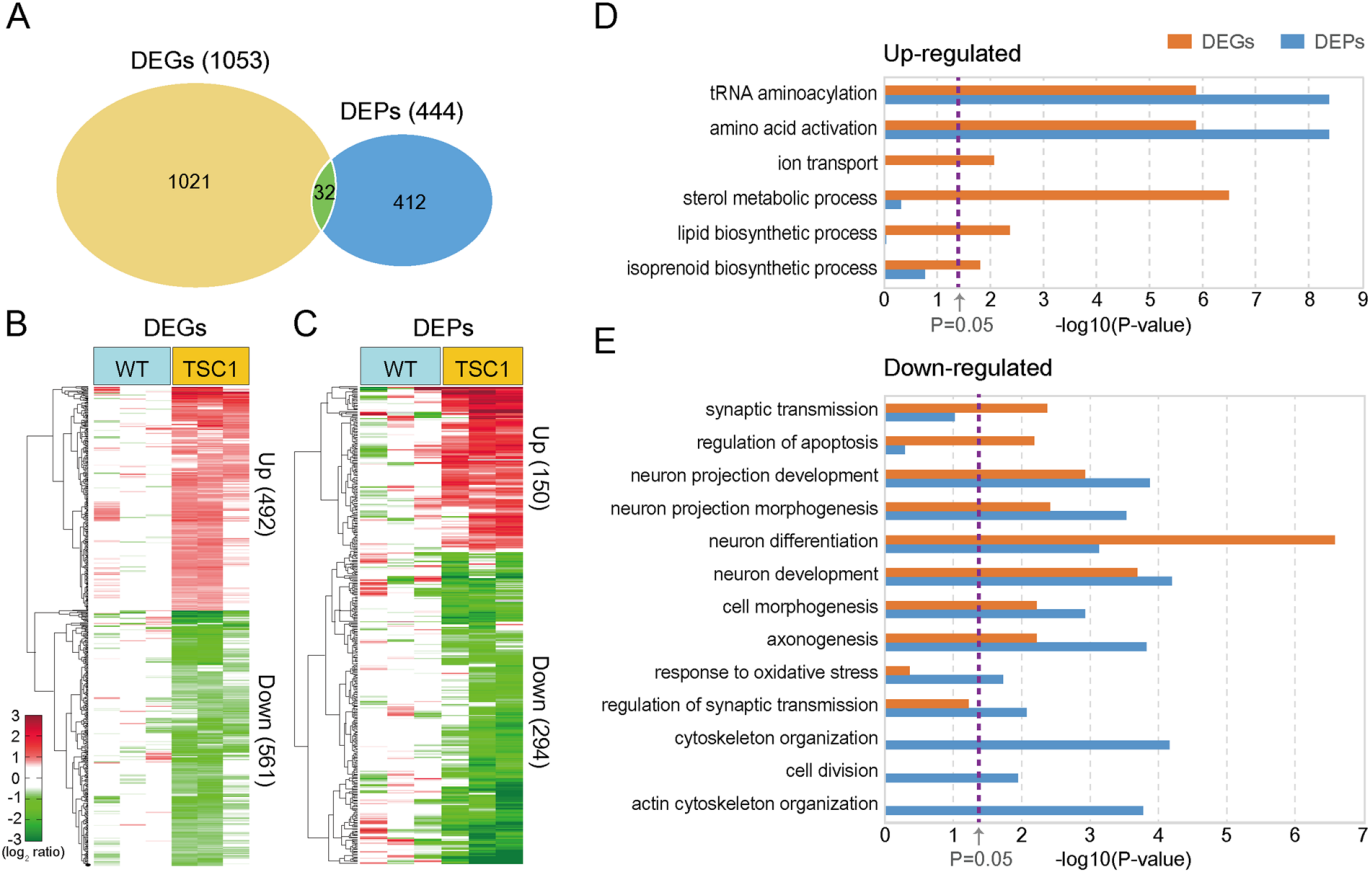
Genes and proteins altered by hyperactivated mTOR signaling. (**A**) Relationships between DEGs and DEPs by hyperactivated mTOR signaling. Numbers in parentheses denote the numbers of DEGs and DEPs. (**B**,**C**) Heat maps showing up- (Up, red) and down-regulation (Down, green) of DEGs (**B**) and DEPs (**C**) between WT (1^st^ to 3^rd^ columns) and TSC1 CKO (4^th^ to 6^th^ columns) samples. The color bar represents gradients of log_2_-fold-changes between TSC1 CKO and WT. Numbers in parentheses denote the numbers of up- and down-regulated genes or proteins. (**D**,**E**) GOBPs represented by up- (**D**) and down-regulated (**E**) genes (orange) or proteins (blue). The significance of GOBPs enriched by DEGs or DEPs was displayed by –log_10_(*P*) where *P* is the enrichment P-value obtained from DAVID software. The color bar denotes gradients of –log_10_(*P*). The cutoff of P-value (P = 0.05) was indicated by the dotted line.

To identify the genes affected by hyperactivated mTOR signalling among the 14,396 expressed genes, we compared their expression levels between TSC1 CKO and WT samples (TSC1 versus WT). From this comparison, we identified 1,053 differentially expressed genes (DEGs; P < 0.05) between TSC1 and WT (Fig. 2A, left) using the integrative statistical method previously reported^11^. The DEGs are comprised of 492 up-regulated and 561 down-regulated genes in TSC1, compared to WT (Fig. 2B; Supplementary Table 1; Materials and Methods). Next, to identify the proteins affected by hyperactivated mTOR signaling, we first quantitated the abundances of the 50,533 peptides from the 4,404 proteins detected by LC-MS/MS analysis based on the intensities of their corresponding UMCs^12^. We then compared the peptide abundances between TSC1 and WT (TSC1 versus WT) and identified 4,989 differentially expressed peptides (DEpeptides; P < 0.05) using the above integrative statistical method^11^. Using the DEpeptides, we finally selected 444 differentially expressed proteins (DEPs) having more than two unique DEpeptides with the same direction of abundance changes (Fig. 2A, right). The DEPs include 150 up-regulated and 294 down-regulated proteins in TSC1 compared to WT (Fig. 2C; Supplementary Table 2; Materials and Methods).

### Cellular processes affected by hyperactivated mTOR signaling

Between the 14,396 expressed genes and the 4,404 detected proteins, 4,137 were common (Fig. 1D). Interestingly, however, only 32 out of 4,137 molecules (0.78%) were found among both the genes (DEGs) and proteins (DEPs) affected by hyperactivated mTOR signaling (Fig. 2A). This low percentage is possibly due to the high level of heterogeneity in molecular signatures targeted by mTOR signaling (see Discussion). Several studies have convincingly demonstrated that cellular process- or pathway-based analysis, despite the frequently seen heterogeneity, can successfully identify key processes or pathways underlying disease pathogenesis utilizing different types of omics data (e.g., transcriptomic and proteomic data)^13–16^. For the cellular process-based analysis, we thus carried out an enrichment analysis of Gene Ontology Biological Processes (GOBPs) associated with the DEGs and DEPs (Supplementary Table 3; Materials and Methods). First, the up-regulated genes and proteins were commonly associated significantly (P < 0.05) with translation-related processes (tRNA aminoacylation and amino acid activation) (Fig. 2D). Second, the down-regulated genes and proteins were commonly associated with neuronal morphogenesis (neuron projection morphogenesis, cell morphogenesis, and axonogenesis) and neuronal development (neuron differentiation and development and neuron projection development) (Fig. 2E), consistent with the developmental and morphological defects in brains previously reported in a mouse model with hyperactivated mTOR signaling^4, 5^. Moreover, cytoskeleton organization enriched by the down-regulated proteins also supports the association of hyperactivated mTOR signaling with the morphological defects in brains. Furthermore, synaptic transmission-related processes were commonly enriched by both the down-regulated genes (synaptic transmission) and proteins (regulation of synaptic transmission), consistent with decreased neuronal synaptic activities previously known to be associated with hyperactivated mTOR signaling^6, 8^. In addition, apoptosis was uniquely enriched by the down-regulated genes, whereas oxidative stress response was uniquely enriched by the down-regulated proteins. Alterations in these two processes may be causally related with the early death previously reported in the mouse model with hyperactivated mTOR signaling^5^. In sum, the significant common associations of the DEGs and DEPs with developmental and morphological processes whose defects are the primary phenotypes seen in the mouse model strongly suggest the validity of the DEGs and DEPs, despite the small overlap between them.

### TSC-implicated cellular processes altered by hyperactivated mTOR signaling

**Figure 3:**
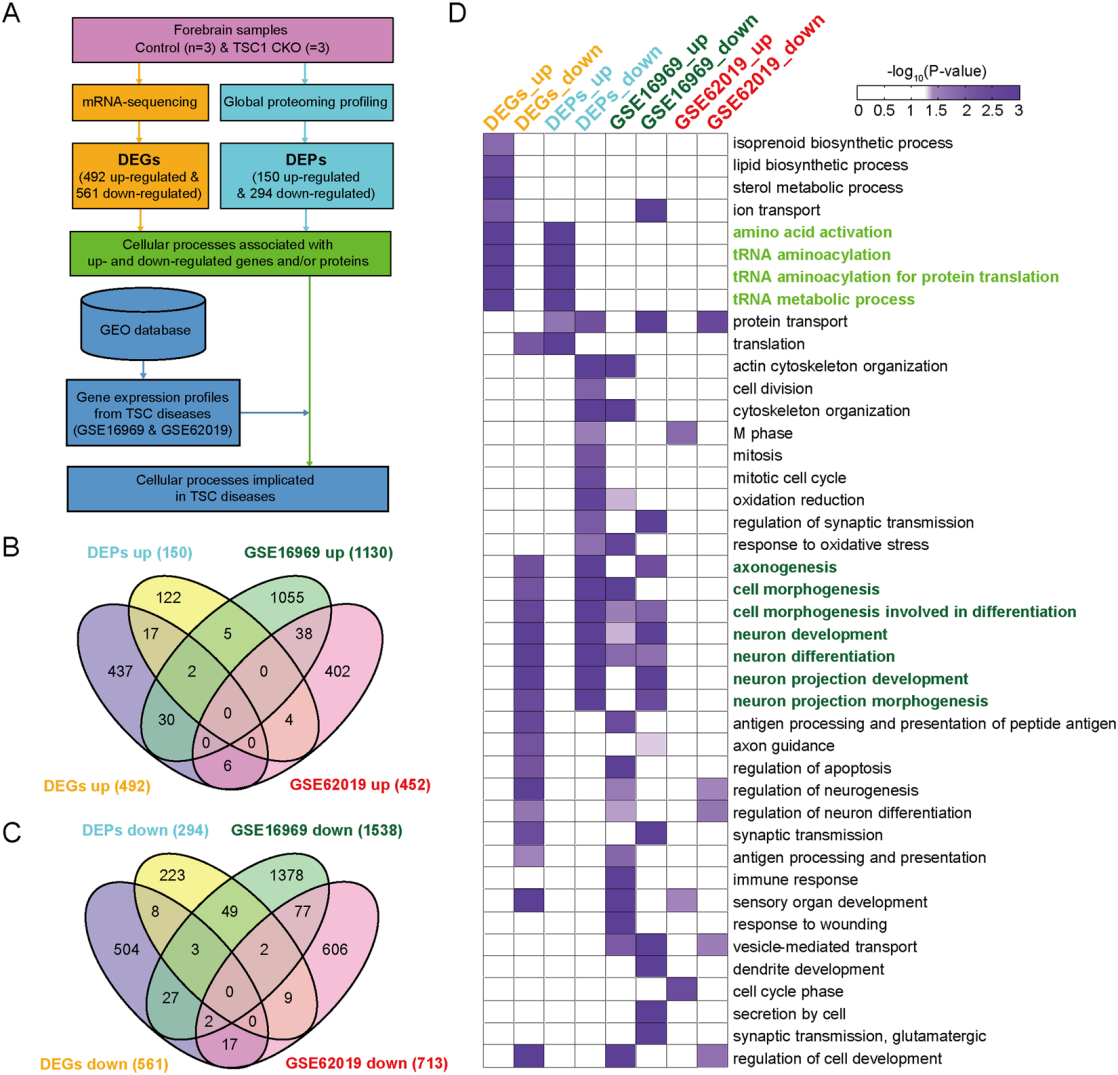
TSC-implicated cellular processes affected by hyperactivated mTOR signaling. (**A**) The overall scheme of integrative analysis of DEGs and DEPs by hyperactivated mTOR signaling and DEGs identified from transcriptomic data of TSC patients (GSE16969 and GSE62019). GOBPs represented by DEGs and DEPs by hyperactivated mTOR signaling and DEGs identified from TSC transcriptomic data were integrated. (**B**,**C**) Relationships among DEGs and DEPs by hyperactivated mTOR signaling and DEGs identified from GSE16969 and GSE62019. Venn diagrams were separately made for up- (**B**) and down-regulated (**C**) genes or proteins. *D*, GOBPs represented by the following eight sets of genes and proteins: 1–4) up- (DEGs_up and DEPs_up) and down-regulated (DEGs_down and DEPs_down) genes and proteins by hyperactivated mTOR signaling and 5–8) up- (GSE16969_up and GSE62019_up) and down-regulated (GSE16969_down and GSE62019_down) genes in the two transcriptomic datasets. The significance of GOBPs being enriched by the indicated sets of genes or proteins was displayed by –log_10_(*P*) where *P* is the enrichment P-value obtained from DAVID software. The color bar denotes gradients of –log_10_(*P*).

The GOBP enrichment analysis showed that a number of cellular processes affected by hyperactivated mTOR signaling were closely associated with developmental and dendritic defects observed in the mouse model of TSC diseases. To further sort out cellular processes with clinical implications, we integrated gene expression profiles obtained from TSC patients with the DEGs and DEPs identified from our mRNA-sequencing and proteomic data (Fig. 3A). First, we obtained gene expression profiles of (1) cortical tubers from 4 TSC patients and 4 autopsy control tissues (GSE16969^17^); and (2) brain tissues from 3 TSC patients and 3 normal brain tissues (GSE62019). For each dataset, we identified the following DEGs using the above integrative statistical method^11^: (1) 2,668 DEGs (1,130 up-regulated and 1,538 down-regulated) for GSE16969 (Fig. 3B) and (2) 1,165 DEGs (452 up-regulated and 713 down-regulated) for GSE62019 (Fig. 3C). These DEGs showed no significant overlaps with our DEGs and DEPs: (1) 37 up-regulated and 83 down-regulated genes identified from GSE16969 and (2) 10 up-regulated and 30 down-regulated genes identified from GSE62019 overlapped with our DEGs and DEPs (Fig. 3B and C, respectively; Supplementary Tables 1 and 2). Due to these small overlaps among the DEGs, as in the case of the DEGs and DEPs, we chose to apply the aforementioned cellular process-based analysis to DEGs from the patient data. To this end, we carried out the GOBP enrichment analysis for up- and down-regulated genes from GSE16969 and GSE62019 and subsequently examined whether any cellular processes were enriched in common with DEGs and DEPs from our mouse model (Supplementary Table 3). Of the GOBPs enriched by both our DEGs and DEPs, the processes related to neuron morphogenesis (axonogenesis, cell morphogenesis involved in differentiation, and neuron projection morphogenesis) and neuron development (neuron development and differentiation and neuron projection development) were enriched by the DEGs from the patient data (dark green-labeled GOBPs in Fig. 3D). In contrast, translation-related processes (amino acid activation, tRNA aminoacylation for protein translation, and tRNA metabolic process) were not enriched by the DEGs from the patient data (light green-labeled GOBPs in Fig. 3D). These data suggest the GOBPs related to neuron morphogenesis and development commonly enriched by our data and patient data as the key downstream events underlying developmental and morphological defects in murine model and TSC patients.

### Network model linking hyperactivated mTOR signaling to developmental and morphological defects in the telencephalon

**Figure 4:**
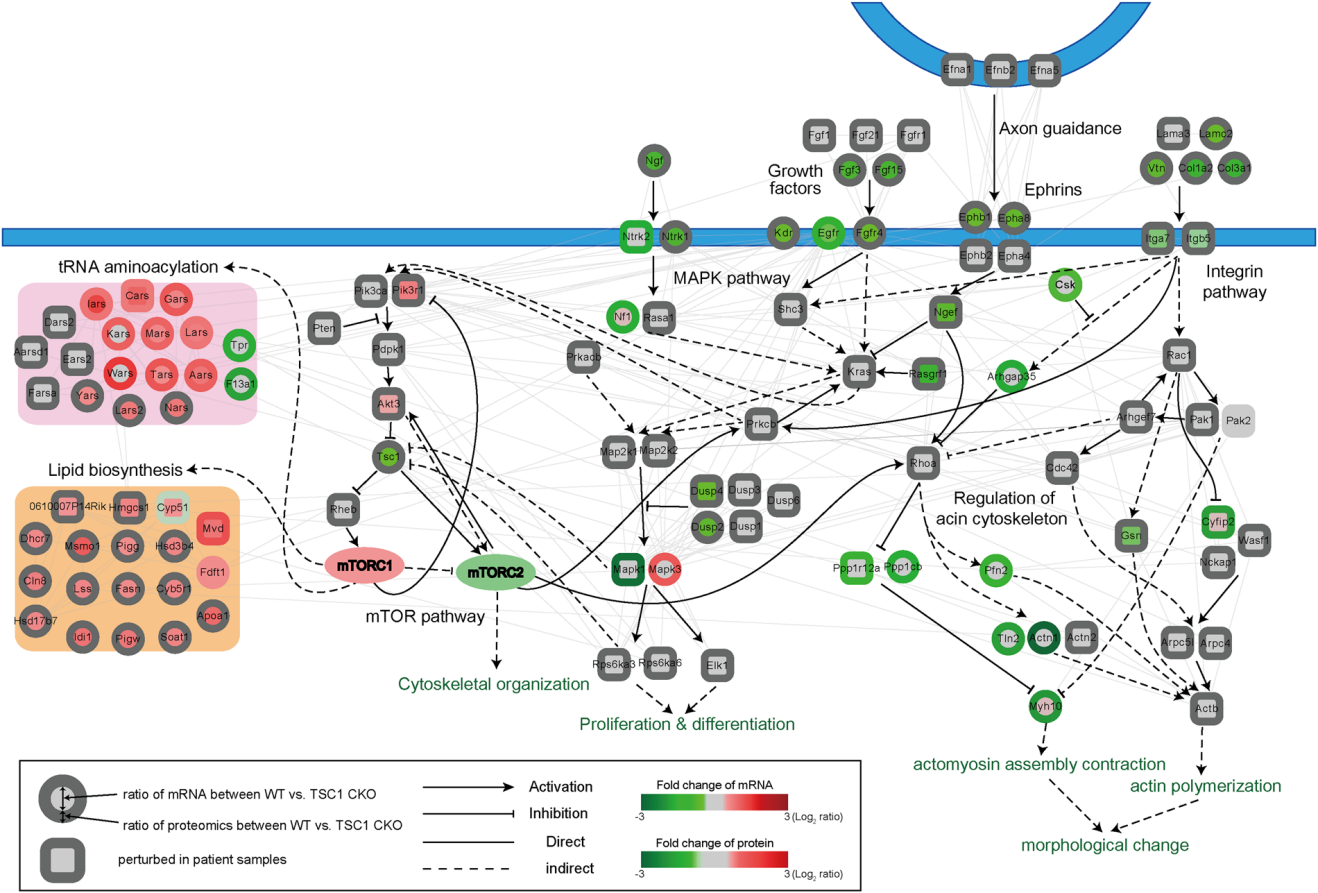
Network model describing association of hyperactivated mTOR signaling with developmental and morphological defects in forebrains. Node colors represent up- (red) and down-regulation (green) of the corresponding genes (center color) and proteins (boundary color) in TSC1 CKO samples compared to WT. The color bar denotes the gradient of log_2_-fold-changes between TSC1 CKO and WT. Edges represent protein-protein interactions (gray) collected from the five interactome databases (Materials and Methods) and activations (arrows) and inhibition (inhibition symbols) obtained from the KEGG pathway database. Solid and dotted lines indicate direct and indirect activation/inhibition, respectively. Plasma membranes are denoted in blue.

To understand collective functions of these key downstream processes in association with the mTOR signaling, we constructed a network model (Fig. 4, right part) describing interactions among down-regulated genes and proteins involved in the processes (dark green-labeled GOBPs in Fig. 3D) related to neuron morphogenesis (axonogenesis, cell morphogenesis involved in differentiation, and neuron projection morphogenesis) and neuron development (neuron development and differentiation and neuron projection development). Importantly, the network model showed that mTOR complex 2 (mTORC2) would be deactivated by TSC1 deletion, and that the mTORC2 interacts with RhoA (Rhoa), MAPK (Map2k1/2 via Prkcb), and Pik3/Akt (Pik3r1/ca via Prkcb) pathways that could modulate the activity of the actin cytoskeleton regulation. Of note, other components in the MAPK (Mapk1, Dusp2/4, and Rasgrf1), RhoA (Arhgap35 and Ppp1r12a/1cb), and Pik3/Akt (Akt3, Isl1, Uchl1, and Pik3r1) pathways were down-regulated by TSC1 deletion, collectively contributing to actin cytoskeleton dysregulation. Down-regulation of MAPK pathway could also lead to aberrant expression of transcription factors such as Elk1 and of neurogenesis-related molecules (Bcl11b, Pbx3, and Dcx) contributing to differentiation and developmental defects in forebrains.

Besides deactivation of mTORC2 and down-regulation of its interacting pathways involved in actin cytoskeleton regulation, the network model also showed that several upstream pathways of actin cytoskeleton regulation were down-regulated by TSC1 deletion, including integrin (Itga5/7), growth factor (Fgf3/15, Fgrf4, Egfr, Ngf, and Ntrk1/2), neurotransmitter (Drd2 and Adora2a), ephrin/netrin (Epha8/b1 and Ntn1), and adhesion (Nrcam) pathways. Moreover, two downstream pathways of actin cytoskeleton regulation, motor complex (Myh10) and actin polymerization (Actn1, Pfn2, and Tln2) pathways, were down-regulated by TSC1 deletion. The convergence of the pathways to regulation of actin cytoskeleton in the network model is consistent with a major role of actin cytoskeleton during neuronal migration and morphogenesis, dendrite formation, and even in learning^18–21^. In particular, down-regulation of the downstream pathways of actin cytoskeleton regulation could be associated with morphological alterations thus providing bases for morphological defects of axon and dendritic spine development.

Among cellular processes involving genes and proteins up-regulated by TSC1 deletion, tRNA aminoacylation and lipid biosynthesis were shown to be associated with mTOR signaling pathway^22, 23^, although they were not enriched by the DEGs from the patient data (Fig. 3D). We thus constructed another network model describing interactions among the DEGs and DEPs involved in tRNA aminoacylation and lipid biosynthesis (Fig. 4, left part) and then combined it with the previous network model for neuron morphogenesis and development. This network model shows that mTOR complex 1 (mTORC1), unlike the mTORC2, would be activated by TSC1 deletion. The network also shows that mTORC1 interacts with tRNA aminoacylation and lipid biosynthesis components, which could lead to up-regulation of protein translation and lipid biosynthesis (see Discussion). Taken together, the combined network model suggests that deactivation of mTORC2, activation of mTORC1, and down-regulation of the pathways interacting with mTORC1 and mTORC2 result in defects in neuronal development and morphogenesis thus explaining the phenotypes seen in the TSC forebrains.

### Validation of mTOR signaling-induced alterations related to developmental and morphological defects in the telencephalon

**Figure 5:**
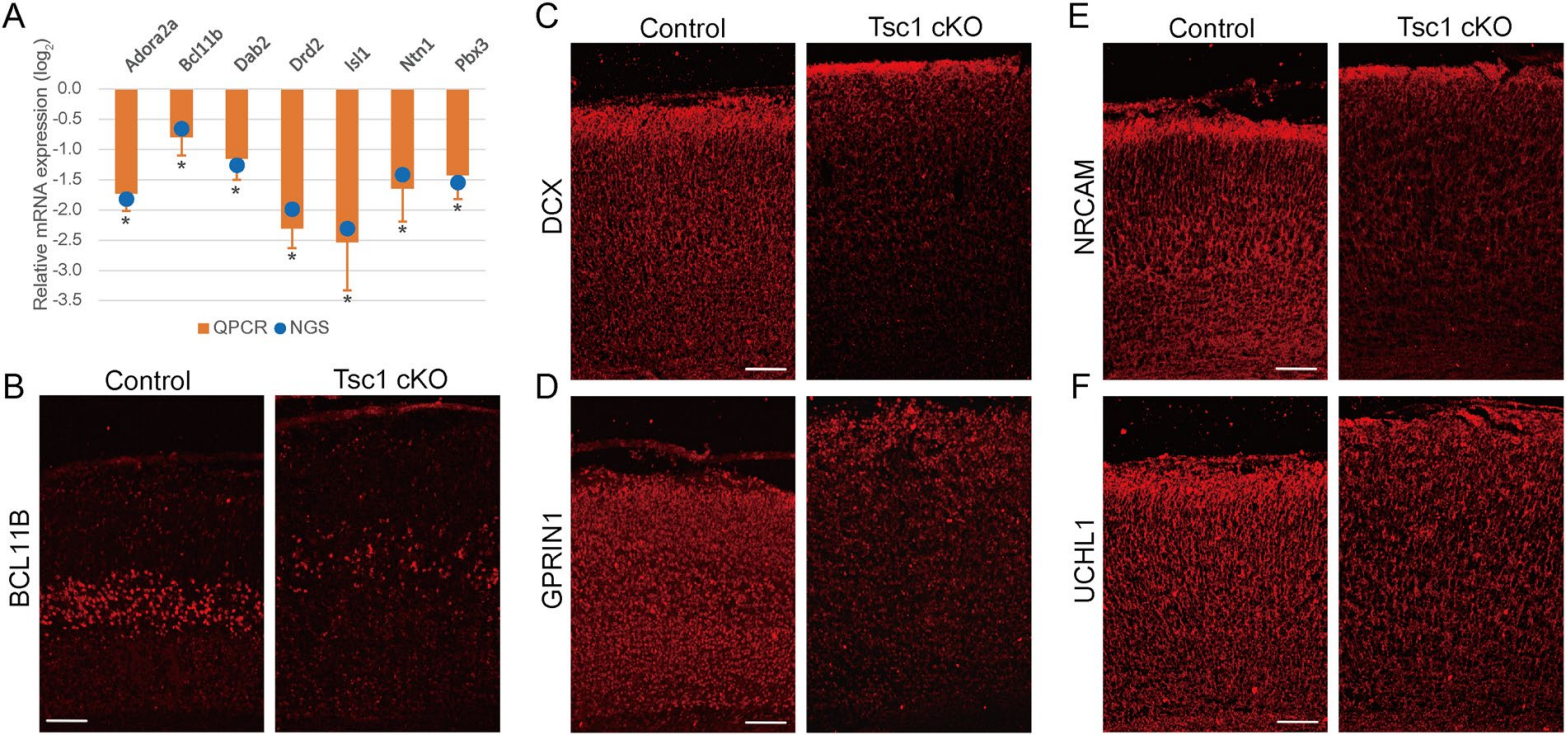
Validation of molecular signatures related to developmental and morphological defects in forebrains. (**A**) Relative mRNA expression levels of seven representative down-regulated genes in TSC1 CKO brain, compared to WT, involved in neuronal morphogenesis and neuronal development are examined using qRT-PCR. *P < 0.05 from Student’s t-test. The blue dots show the expression levels from transcriptomic analysis. (**B**–**F**) Expression of five representative down-regulated proteins involved in neuronal morphogenesis and neuronal development are examined. The results from immunohistochemical staining using WT and TSC1 CKO cortex at P0 are shown. Scale bar = 100 µm.

Finally, we experimentally confirmed the alterations of molecules by hyperactivated mTOR signaling using multiple markers. Representative genes and proteins were selected respectively from DEGs and DEPs involved in the aforementioned cellular pathways related to developmental and morphological defects. First, we confirmed down-regulation of seven representative genes involved in the following cellular pathways, using quantitative real-time polymerase reaction (qRT-PCR) (Fig. 5A): (1) neurotransmitter (Drd2 and Adora2a), (2) netrin (Ntn1), (3) MAPK (Pbx3 and Bcl11b), and (4) Pik3/Akt (Isl1) pathways. An intermediate signaling molecule, Dab2 was also examined. Second, we confirmed down-regulation of five representative proteins involved in the following cellular pathways using immunohistochemistry analysis (Fig. 5B–F): (1) adhesion (Nrcam), (2) MAPK (Bcl11b and Dcx), and (3) Pik3/Akt (Uchl1) pathways. Again, an intermediate signaling molecule, Gprin1 was also examined. Of note, Bcl11b, a transcriptional repressor, showed down-regulation in TSC compared to WT, at both mRNA and protein levels as confirmed by qRT-PCR and immunohistochemistry analyses respectively. These data collectively not only validate DEGs and DEPs, but also confirm in particular the down-regulation of cellular pathways related to developmental and morphological defects shown in the network model (Fig. 4).

## Discussion

Hyperactivation of mTOR signaling has been implicated in diverse developmental and morphological defects in the brain. However, the network of downstream molecules underlying such developmental and morphological defects has been largely unknown. In this study, we systematically explored the downstream molecules at mRNA and protein levels through comprehensive transcriptome and proteome profiling of WT and TSC1 telencephalons. Our integrative analysis of transcriptomic and proteomic data incorporates 1) comprehensive transcriptome and proteome profiling of telencephalon tissues using mRNA-sequencing and label-free LC-MS/MS, respectively; (2) identification of DEGs and DEPs by hyperactivated mTOR signaling and their associated cellular processes; (3) identification of TSC-implicated cellular processes commonly represented by DEGs, DEPs, and also DEGs identified from transcriptome profiles of TSC patients; (4) assignation of molecular signatures representing the cellular pathways related to actin cytoskeleton regulation based on the network model; and (5) validation of alterations of the selected molecular signatures in independent TSC1 samples using qRT-PCR and immunohistochemistry analyses.

Using this approach, we propose a network model illustrating the linkage between hyperactivated mTOR signaling caused by TSC1 deletion and morphological and developmental defects in the telencephalon. The network model shows deactivation of mTORC2 and activation of mTORC1 by TSC1 deletion. The mTORC2 interacts with the pathways (RhoA, MAPK, and Pik3/Akt pathways) involved in actin cytoskeleton regulation potentially contributing to developmental and morphological defects in TSC1-deleted telencephalon. The activated mTORC1 could inhibit the function of mTORC2, thereby facilitating deactivation of mTORC2-dependent actin cytoskeleton regulation. Thus, deactivation of mTORC2 and activation of mTORC1 can serve as two molecular bases that can result in morphological and developmental defects in the TSC1-deleted telencephalon. The network model also showed that the activated mTORC1 interacts with tRNA aminoacylation and lipid biosynthesis, which could result in up-regulation of these two processes. These two processes are associated with the synthesis of proteins and cellular membranes (plasma membrane, membranous organelles, or vesicles), respectively. Although their importance in cellular differentiation is without question, how their up-regulation is associated with specific neuronal defect seen in TSC remains to be resolved down the road.

The tissues collected from animal models or patients can show high levels of heterogeneity in molecular signatures (e.g., mRNA or protein levels), which often leads to a small overlap among dysregulated molecules (e.g., DEGs and DEPs) that were identified from different omics data (e.g., transcriptomic and proteomic data)^24–26^. In our study, after observing small overlaps among DEGs and DEPs from our data and DEGs from patient data (Fig. 3B,C), we applied the cellular process- and pathway-based analyses to identify important cellular processes and pathways dysregulated by hyperactivated mTOR signaling in the forebrain. The analyses revealed that the DEGs and DEPs together with DEGs from patient data were commonly associated with cellular processes (Fig. 3D) and pathways (network model in Fig. 4) involved in neuron morphogenesis and neuron development. Considering that the chance for two different types of omics data (transcriptomic and proteomic data) and two independent sets of patient data implicating common processes and pathways by coincidence is low, the integration of all these different datasets as well as conclusions drawn from the integration appears to be meaningful.

Recently, several proteogenomic analyses have been performed for the Cancer Genome Atlas (TCGA) colorectal, breast, and ovarian cancer samples^27–29^. These studies have shown that the proteogenomic analyses can facilitate prioritization of cancer-associated somatic mutations, identification of reliable cancer subtypes, and elucidation of functional links of genetic alterations to cancer-associated signaling networks. Our integrative analysis of transcriptomic and proteomic data is different from the previous proteogenomic analyses in several aspects. In this study, we have not generated exome-sequencing data with which sequence-level analysis (e.g., mutations) can be carried out. Also, it should be noted however that DEGs and DEPs but not mutations are responsible for the phenotypes of mouse model used in this study. Moreover, with no phosphoproteome data available, we could not analyze the potential linkage between mutations and cellular signaling pathways. Furthermore, in the aforementioned proteogenomic analyses, the correlation between mRNA and protein abundances was determined based on over 100 samples^27–29^. In this study, the transcriptomic and proteomic data were obtained from only six samples (three WT and three TSC1 samples). Despite this limitation, for genes with both mRNA and protein abundance data available, we analyzed the correlation between mRNA expression levels and protein relative abundances. The mean mRNA-protein correlation for all the pairs of gene and protein was found to be 0.07 (Spearman correlation), which was lower than 0.23 (mean Spearman correlation) for colorectal cancer, 0.39 (median Pearson correlation) for breast cancer, and 0.38 (mean Spearman correlation) for ovarian cancer consistent with the small overlap between DEGs and DEPs. In summary, our integrative analysis of transcriptomic and proteomic data provided extensive lists of DEGs and DEPs by hyperactivated mTOR signaling, thus meaningfully extending the current list of TSC-associated molecules and cellular pathways identified by conventional small-scale experiments. These lists should serve as comprehensive resources to those who study functional links of mTOR signaling to the pathogenesis of TSC. Our study also provided the molecular signatures and their associated pathways shown in the network model (Fig. 4), some of which have been previously linked to mTOR signaling in brain. Specifically, GOBP enrichment analysis above showed that a number of cellular processes enriched by up- and down-regulated genes or proteins by hyperactivated mTOR signaling were closely associated with neuronal developmental and dendritic defects observed in the mouse model of TSC diseases. The clinical implications should be further tested with a larger number of TSC patients. In addition, longitudinal studies based on molecular signatures and their associated pathways can be designed *in vitro* or *in vivo* for further understanding of the development-associated nature of TSC pathogenesis. Such successful dissection of the molecular nature of TSC may also facilitate identification of therapeutic options for multiple mTOR signaling-related neurological disorders.

## Methods

### Generation of Tsc1/Emx1-cre CKO mice

Tsc1-floxed mice (strain #005680, Tsc1tm1Djk/J) and Emx1-cre mice (strain #005628, Emx1tm1(cre)Krj/J) were obtained from The Jackson Laboratory. Through interbreeding, Tsc1flox/flox;Emx1-cre/+ (Tsc1/Emx1-cre) conditional knockout (CKO) mice were generated, whose exons 17 and 18 of Tsc1 gene were deleted by cre recombinase expressed in embryonic dorsal cortex. Homozygous Tsc1-floxed;Emx1-cre negative littermates were used as controls. Tail genomic DNA was extracted using lysis buffer (0.025 N NaOH, 0.1 mM EDTA and 2.5% Triton X-100), and genotyping was performed by PCR with primers (floxed-Tsc1: 5′-AGGAGGCCTCTTCT GCTACC-3′ and 5′- CAGCTCCGACCATGAAGTG-3′, Emx1-cre: 5′-GCATTACCGGTCG ATGCAACGAGTGATGAG-3′ and 5′-GAGTGAACGAACCTGGTCGAAATCAGTGCG-3′). All experimental procedures were reviewed and approved by the Institutional Animal Research Ethics Committee of Ajou University Medical Center (South Korea) and performed in accordance with the relevant guidelines and regulations.

### mRNA-sequencing experiments

Total RNAs were extracted from telencephalon tissues of Tsc1/Emx1-cre CKO and control mice at P0 with the TRIzol reagent (Ambion) according to the standard procedure. Briefly, the tissue specimens were mechanically homogenized in TRIzol reagents with chloroform later added. After vortexing, the samples were incubated for 5 min at room temperature and centrifuged for 15 min with 14,000 rpm at 4 °C. The colorless upper phase was precipitated by mixing with the same volume of isopropanol and incubating for 10 min at room temperature. After centrifugation for 15 min with 14,000 rpm at 4 °C, the pellet was washed with 70% ethanol and air-dried for 10 min. Finally, the RNA was dissolved in DepC-water (Ambion). The quality of RNA was measured using Agilent Bioanalyzer 2100 (Agilent) and all samples showed RNA integrity number (RIN) >7. The quantity of RNA was assessed by NanoDrop (ND-1000) Spectrophotometer (Thermo Scientific). Total RNA libraries were prepared using Illumina Truseq RNA prep kit v2 according to the manufacturer’s standard protocol. RNA sequencing was performed on Illumina HiSeq 2500 platform using 101 bp paired-end runs. Sequencing reads were aligned to the mouse genome (mm10 from the UCSC genome database) using Mapsplice2 (version 2.1.7) after standard quality check and trimming with FastQC and Fastx-toolkit (version 0.0.13.2), respectively. RSEM (version 1.2.12) was used to quantify the mRNA abundance.

### Identification of differentially expressed genes

We identified differentially expressed genes (DEGs) between Tsc1/Emx1-cre CKO and control mice using the previously reported statistical method^30^. Briefly, *T* value and log_2_-median-ratio were computed for each gene. An empirical distribution of the null hypothesis (i.e., a gene is not differentially expressed) was estimated by calculating *T* values and log_2_-median ratios for the genes after randomly permuting the samples. For each gene, the adjusted *P*-values of the observed *T* value and log_2_-median ratio were computed using their corresponding empirical distributions by two-tailed tests and then combined into an overall *P* value using Stouffer’s method^31^. The DEGs were selected as the genes with 1) overall *P*-values < 0.05 and (2) absolute log_2_-fold-changes ≥95^th^ percentile (0.42) of the empirical null distribution of log_2_-median-ratio.

### Label-free peptide quantification

Detailed descriptions of protein preparation, MS analysis, and peptide identification are provided as supplementary methods. To assign MS intensity to peptide identification, an MS intensity-based label-free quantification method was applied to 24 LC-MS/MS datasets as described previously^32^. Briefly, through PE-MMR analysis, MS features of a peptide that appeared over a period of LC elution time in an LC-MS/MS experiment were grouped into a unique mass class (UMC)^33^. Ideally, each UMC corresponds to a peptide and contains the ions corresponding to the peptide, together with their mass spectral features, such as charge states, abundances (intensity), scan numbers, and measured monoisotopic masses. For each UMC, we obtained the mass by calculating the intensity-weighted average of the monoisotopic masses of all the ions in the UMC. For the peptide corresponding to the UMC, its abundance was estimated as the summation of the abundances of all the ions in the UMC (UMC intensity). The refined precursor masses of the MS/MS spectra by PE-MMR were matched to the UMC masses and then were replaced with those of UMCs. In this process, MS/MS spectra information was linked to the matched UMC. The linked MS/MS spectra were assigned with a peptide sequence (peptide ID) with a FDR ≤ 0.01 after MS-GF+ searching and target-decoy analysis. The peptide ID was assigned to the UMC, and the UMC intensity was assigned to the peptide ID.

### Assignment of UMCs by master AMT database

To assign the peptide IDs to unidentified UMCs, the master accurate mass and time tag (AMT) database (DB) was constructed and utilized as described previously^9^. Briefly, the information about UMCs with peptide IDs from 24 LC-MS/MS data (triplicate LC-MS/MS experiments of 8 samples) was compiled into the master AMT-DB. The AMTs are unique peptide sequences whose monoisotopic masses and normalized elution times (NETs^34^); are experimentally determined. For each AMT whose corresponding peptides were measured multiple times, the average mass and the median NET were recorded. We then mapped unidentified UMCs to AMTs in the master AMT DB with ±10 ppm of mass and ±0.02 of NET tolerances, and 3.0 of Excorr threshold. For each unidentified UMC matched to an AMT, the peptide ID for the AMT was assigned together with all information for the AMT (UMC mass, NET and Excorr).

### Alignment of the identified peptides

After the assignment of unidentified UMCs using the master AMT DB, we combined the assigned and identified UMCs from LC-MS/MS datasets into an *n* $\times \,$ *m* alignment table (peptide IDs for *n* UMCs and UMC intensities in *m* samples). The missing value in the alignment table means that the peptide was not identified in the corresponding datasets. For the missing values in each row of the table, we further searched for the UMCs that could be matched to the aligned UMCs based on their UMC masses and NETs with the following tolerance: ±10 ppm and ±0.01 across the technical replicates, and ±10 ppm and ±0.02 across the biological replicates. Quantile normalization was performed for UMC intensities in the alignment table to correct systematic variations of peptide abundance across datasets^35^. To evaluate reproducibility of LC-MS/MS analysis, two types of similarity scores between LC-MS/MS datasets were calculated by measuring the overlap of detected peptides and their intensity values, as previously described^36^.

### Identification of DEPs

To identify DEPs, we fist selected differentially expressed peptides (DEpeptides) by applying a previously reported integrative statistical method^30^ to UMC (peptide) intensities in the alignment table. Briefly, log_2_-intensities of each peptide in TSC1 samples were compared to those in control samples by Student’s t-test and median-ratio test, which resulted in *T* value and log_2_-median ratio respectively for each peptide. We then estimated empirical null distributions of *T* values and log_2_-median ratios by randomly permuting 18 samples 1,000 times. For each peptide, the adjusted *P* values of the observed *T* value and log_2_-median ratio were computed using the empirical distributions with two-tailed test and then integrated into an overall *P* value by using Stouffer’s method^31^. The DEpeptides were identified as the peptides with their overall *P* < 0.05 and absolute log_2_-fold-change ≥0.58 (1.5-fold in the original scale). Finally, the DEPs were identified as the proteins with the numbers of up- or down-regulated unique DEpeptides ≥2.

### Functional enrichment analysis of DEGs or DEPs

The enrichment analysis of Gene Ontology Biological Processes (GOBPs) for a list of proteins (up- and down-regulated genes or proteins) was performed using DAVID software^37^. GOBPs represented by the list of genes or proteins were identified as those with *P* < 0.05.

### Reconstruction of a network model

To reconstruct a network model for cellular processes and pathways affected by hyperactivated mTOR signaling, we first selected a subset of DEGs or DEPs involved in GOBPs related to the aforementioned developmental and morphological defects (dark green-labeled GOBPs in Fig. 3D) caused by hyperactivated mTOR signaling. Also, of the interactors of the selected genes and proteins, we added to the network model those that overlapped with our DEGs/DEPs or DEGs from TS data. Next, for the selected genes/proteins and the interactors, we collected protein-protein interactions obtained from six interactome databases: the Biological General Repository for Interaction Datasets (BioGRID)^38^, the Database of Interacting Proteins (DIP)^39^, High confidence protein-protein interactions (HitPredict)^40^, the IntAct molecular interaction database (IntAct)^41^, the Molecular INTeraction database (MINT)^42^, and functional protein association networks (STRING)^43^. Finally, we built a network model describing interactions among the selected genes/proteins and the interactors using Cytoscape^44^. The nodes in the network model are arranged into intracellular signaling pathways based on the information in Kyoto encyclopedia of genes and genomes (KEGG) pathway databases^45^.

### qRT-PCR analysis

Total RNA preparations (1 μg) obtained from telencephalon of Tsc1/Emx1-cre CKO and control mice at P0 were reverse transcribed into cDNAs using QuantiTect Reverse Transcription Kit (QIAGEN) according to the manufacturer’s instruction. qRT-PCR was run on a CFX96TM real time system (BIORAD) using Power SYBR^®^ Green Master Mix (Applied Biosystems). Samples were prepared in duplicates, and primer pairs are listed in Supplementary Table 4. All values were normalized to mouse β-actin and then standardized to the control condition. Error bars represent S.E.M., and the statistical significance was assessed using the Student’s t-test (*P < 0.05).

### Immunohistochemistry analysis

Mouse brains were fixed in 4% paraformaldehyde overnight at 4 °C, cryo-protected with 30% sucrose in PBS, embedded in O.C.T. compound (Scigen Scientific Gardena) and sectioned at 16 μm thickness using Microtome cryostat (Microm, HM520). The sections were treated with PBS containing 5% goat serum and 0.1% Triton X-100 for 1 hr at room temperature and incubated overnight at 4 °C with primary antibodies listed in Supplementary Table 5. The secondary antibodies conjugated to Alexa Fluor^®^ 488 and 594 dyes (Invitrogen) were used at a dilution of 1:300–500 for 1 hr. Images were obtained using a Zeiss LSM510 META Confocal Scanning Laser microscope (Carl Zeiss).

## Electronic supplementary material

Supplementary infromation

Supplementary table 1

Supplementary table 2

Supplementary table 3
