# Supplementary material for: Characterization of developmental defects in the forebrain resulting from hyperactivated mTOR signaling by integrative analysis of transcriptomic and proteomic data
Source: Sci Rep. 2017 Jun 6;7:2826. doi: 10.1038/s41598-017-02842-6 (PMC5460284; doi:10.1038/s41598-017-02842-6)
Supplement: Supplementary file 1 — Supplementary infromation [file 41598_2017_2842_MOESM1_ESM.pdf]

Characterization of developmental defects in the forebrain resulting from hyperactivated mTOR signaling by integrative analysis of transcriptomic and proteomic data

Jiheon Shin[1,2,*], Minhyung Kim[3,*], Hee-Jung Jung[4,*], Hye Lim Cha[5], Haeyoung Suh-Kim[5,6], Sanghyun Ahn[4], Jaehoon Jung[4], YounAh Kim[4], Yukyung Jun[1,2], Sanghyuk Lee[1,2,8], Daehee Hwang[3,4,7,#] and Jaesang Kim[1,2,8#].

This document includes:

Supplementary Methods

Supplementary Tables 1-5

Supplementary methods

Protein extraction and digestion

The telencephalon tissue samples were dried and then homogenized with the lysis buffer [urea, NaCl, 50 mM Tris-HCl (pH 8.2) and a Complete Mini Protease inhibitor tablet (Roche Applied Science, Basel, Switzerland)]. The lysate was ultracentrifuged at 45,000 *g* at 4 ℃ for an hour. Protein concentration was determined from the resulting supernatant by DC protein assay (Bio-Rad, Hercules, USA). The protein sample was then reduced in 0.5 M DTT (100 µl) for 50 minutes at 37 ℃, followed by addition of 1.5 µl of 1M iodoacetamide and incubation in the dark for 30 minutes at room temperature for alkylation. The resulting sample was subjected to in-solution tryptic digestion [1:50 enzyme-to-protein ratio (w/w), Promega, Madison, USA] with incubation at 37 ℃ overnight. The peptide samples were centrifuged at 2,500 *g* for 10 minutes at room temperature, and the resulting aqueous solution was desalted using solid-phase extraction with a reverse-phase tC18 SepPak solid-phase extraction cartridge as previously described[1]. Finally, peptides were eluted with 50% ACN 0.5% HAcO and then dried in a Speed-Vac with resuspension in 0.1% formic acid. The samples were stored at -20 ℃ before LC-MS/MS analysis.

LC-MS/MS analysis

All peptide samples were separated on a Thermo EASY-nLC 1000 (Thermo Scientific, Odense, Denmark)[2] equipped with an analytical column (Thermo Scientific, Easy-Column, 75 µm × 50 cm) and a trap column (75 µm × 2 cm). The operation temperature of the analytical column was at 50 ℃. The flow rate was set to 300 nL/min. The solvents A and B were 0.1% formic acid and 2% acetonitrile in water and 0.1% formic acid and 2% water in acetonitrile,

respectively. For the global proteome analysis, 120 min gradient (from 2% to 40% solvent B over 90 min, from 40% to 80% solvent B over 10 min, 80% solvent B for 10 min, and from 80% to 2% solvent B over 10 min) was used. The eluted peptides from LC were analyzed using Q-Exactive™ hybrid quadrupole-Orbitrap mass spectrometer (Thermo Scientific)[3] equipped with a nanoelectrospray source. For the ionization of the eluted peptides, the electric potential of electrospray ionization was kept at 1.7 kV, and the temperature of desolvation capillary was set to 270 ℃. Q-Exactive was operated in the data dependent mode with survey scans acquired for the mass range of 450-2000 Thomsons (Th) at a resolution of 70,000 (at m/z 200). Up to the top 10 most abundant ions from the survey scan were selected with an isolation window of 2.0 Th and fragmented by higher energy collisional dissociation (HCD)[4] with normalized collision energies of 25 and the exclusion duration of 10 s. The MS/MS scans were acquired at a resolution of 17,500. Maximum ion injection times were 100 ms and 50 ms for full MS and MS/MS scan, respectively. Automated gain control (AGC) target value was set to 1.0×10^6 and 1.0×10^5 for full MS and MS/MS scan, respectively.

Peptide identification

The fragmentation spectra were created based on mzXML file using msconvert (ProteoWizard release: 3.0.4323). MS data were first analyzed using post-experiment monoisotopic mass refinement (PE-MMR) to assign accurate precursor mass to tandem MS data[5]. MS/MS spectra were searched against a composite database containing Swiss-Prot entries of the mouse reference proteome (UniProtKB release 2016_02, 25,010 entries) and 179 common contaminants in the target-decoy setting using MS-GF+ Beta (v10089) search engine[6] under the following parameters: semi-tryptic, precursor mass tolerance of 10 ppm, carbamidomethylation of cysteine as a fixed modification, and oxidation of methionine as a

variable modification. The search results of 24 LC-MS/MS datasets were all combined, and the target-decoy analysis was performed on the combined dataset to obtain peptides with the false discovery rate (FDR) ≤ 0.01.

Supplementary Table 1. List of differentially expressed genes (DEGs) between TSC1 CKO and WT. For each DEG, Entrez ID, HUGO gene symbol, and gene description are shown together with up- or down-regulation status in TSC1 compared to WT, P-value, and $\log_2$-fold-change. Also, whether each DEG is up- or down-regulated in TSC patients from GSE16969 and GSE62019 datasets is also indicated.

See the attached excel file titled "Supplementary Table 1.xlsx"

Supplementary Table 2. List of differentially expressed proteins (DEPs) between TSC1 CKO and WT. For each DEP, Entrez ID, HUGO gene symbol, and description are shown together with up- or down-regulation status in TSC1 compared to WT, P-value, and $\log_2$-fold-change. Also, whether each DEP is up- or down-regulated in TSC patients from GSE16969 and GSE62019 datasets is also indicated.

See the attached excel file named "Supplementary Table 2.xlsx"

Supplementary Table 3. Gene Ontology Biological Processes (GOBPs) enriched by DEGs, and DEPs, as well as DEGs identified from gene expression data of TSC patients. For each GOBP, the enrichment information for each of the eight sets of genes shown in Fig. 3D is provided: 1) up-regulated genes (DEG_up), 2) down-regulated genes (DEG_down), 3) up-regulated proteins (DEP_up), 4) down-regulated proteins (DEP_down), 5) up-regulated genes

in GSE16969 dataset (GSE16969_up), 6) down-regulated genes in GSE16969 dataset (GSE16969_down), 7) up-regulated genes in GSE62019 dataset (GSE62019_up), and 8) down-regulated genes in GSE62019 dataset (GSE62019_down). For each GOBP, the enrichment information for each set of genes includes 1) the number of genes annotated with the GOBP in the gene set (Gene_count), 2) the enrichment P-value, whether the GOBP was significantly ($P < 0.05$ and Gene_count ≥ 4) enriched or not (GOBP_enrich), and 4) the list of genes annotated with the GOBP in the gene set. The GOBPs in yellow background are displayed in Fig. 3D.

See the attached excel file named “Supplementary Table 3.xlsx”

Supplementary Table 4. List of primers used for qRT-PCR analysis.

Gene	Primer sequence (5'→3')	
b-actin	forward	GGCTACAGCTTCACCACCACA
	reverse	CGCTCGTTGCCAATAGTGATG
Adora2a	forward	CCATTCGCCATCACCATCAG
	reverse	CTCATACCCGTCACCAAGCC
Bcl11b	forward	TGATTACTTCACCTCTGCGTGC
	reverse	GTGCAAATGTAGCTGGAAGGC
Dab2	forward	GGCAACAGGCTGAACCATTAG
	reverse	ATCCATCTGGTCAACACCCAG
Drd2	forward	CCATTGTCTGGGTCCTGTCC
	reverse	TAGACCAGCAGGGTGACGATG
Isl1	forward	AGCAGCAGCAACCCAACG
	reverse	AAGCAGGCTGATCTATGTCGC
Ntn1	forward	GGCTTCTACCGAGACATGGG
	reverse	TGGTAGCCTTTGGCACATCG
Pbx3	forward	TTACCAAGGGTCCCAAGTCG
	reverse	GAAGATGGAGTTGTTGCGTCC

Supplementary Table 5. List of antibodies used for immunohistochemistry analysis.

Antigen	Host	Manufacturer	Cat.No.	Dilution
BCL11B	Rat	Abcam	ab18465	1:500
DCX	Rabbit	Abcam	ab18723	1:500
GPRIN1	Rabbit	Abcam	ab203632	1:100
NRCAM	Rabbit	Abcam	ab24344	1:300
UCHL1	Rabbit	Novus Biologicals	NB300-676	1:100

References

1 Villen, J. & Gygi, S. P. The SCX/IMAC enrichment approach for global phosphorylation analysis by mass spectrometry. *Nature protocols* **3**, 1630-1638, doi:10.1038/nprot.2008.150 (2008).

2 Nagaraj, N. *et al.* System-wide perturbation analysis with nearly complete coverage of the yeast proteome by single-shot ultra HPLC runs on a bench top Orbitrap. *Molecular & cellular proteomics : MCP* **11**, M111 013722, doi:10.1074/mcp.M111.013722 (2012).

3 Michalski, A. *et al.* Mass spectrometry-based proteomics using Q Exactive, a high-performance benchtop quadrupole Orbitrap mass spectrometer. *Molecular & cellular proteomics : MCP* **10**, M111 011015, doi:10.1074/mcp.M111.011015 (2011).

4 Olsen, J. V. *et al.* Higher-energy C-trap dissociation for peptide modification analysis. *Nature methods* **4**, 709-712, doi:10.1038/nmeth1060 (2007).

5 Shin, B. *et al.* Postexperiment monoisotopic mass filtering and refinement (PE-MMR) of tandem mass spectrometric data increases accuracy of peptide identification in LC/MS/MS. *Molecular & cellular proteomics : MCP* **7**, 1124-1134, doi:10.1074/mcp.M700419-MCP200 (2008).

6 Kim, S. & Pevzner, P. A. MS-GF+ makes progress towards a universal database search tool for proteomics. *Nature communications* **5**, 5277, doi:10.1038/ncomms6277 (2014).
